# Epizootic Suppurative Cellulitis; Or, Phlegmonous Erysipelas in Horses*Abstract of a clinical lecture recently delivered at Columbia Veterinary College.

**Published:** 1882-01

**Authors:** George H. Berns


### ART. III.—EPIZOOTIC SUPPURATIVE CELLULITIS; OR, PHLEGMONOUS ERYSIPELAS IN HORSES.*

BY GEORGE H. BERNS, D.V.S.

While a great deal has been published in the daily papers, agricultural and medical journals on the subject of epizootic cellulitis, catarrhal influenza, or pink-eye, as the late epizootic disease among our horses is popularly termed, nothing has been said upon *Suppurative Cellulitis*, which, in my opinion, is more important, more difficult to treat, and far more dangerous than the ordinary pink-eye.

It consists of an acute inflammation of the subcutaneous cellular connective tissues, which terminates in suppuration and destruction not only of the connective tissues involved, but generally in devitalization of the skin that covers the affected parts, and in some instances the sheaths of tendons, coats of blood-vessels, nerves and synovial membranes become affected, and cause a fatal termination from open joints, blood-poisoning, sympathetic fever, or general prostration. I venture to say that at least fifty per cent. of all the horses in Brooklyn have suffered to a greater or less extent from the ordinary non-suppurative form of cellulitis during the last two or three months, and it is a well-known fact, that the disease ran its regular course, and in very nearly all uncomplicated cases, terminates in restoration to perfect health in from five to fifteen days.

Last fall and winter, immediately after the epizooty, and again this fall a large number of horses are suffering from a very unmanageable form of inflammation of the skin, a subcutatious areolar tissue of the pastern and fetlocks, which in aggravated cases extending all the way up the limb to the thigh, or elbows. This inflammation appears to originate in the cellular connective tissue, and terminates in suppuration and the formation of large abscesses, constituting what is known as *Suppurative Cellulitis*.

Last season this disease was considered by many to be a malignant form of *Erythema*, *Mud Fever*, or *Scratches*, due to the filthy condition of the streets, and improper attention to the

*Abstract of a clinical lecture recently delivered at Columbia Veterinary College.

horses feet and legs on the part of their attendants; others believed it to be *frost bite* of the heels and pasterns, and blamed the railroad companies for throwing salt on their tracks. That these views are erroneous, is evident from the fact that the same disease prevails this season, which has been remarkably dry and mild.

I believe this local form of cellulitis to be due to the same obscure and unknown causes which produced the late epizooty, and at this time I will not venture into any speculative theories as to the probable causes of that disorder, for nothing is positively known upon the subject.

*Symptoms.*—We are called upon to see a lame horse, and are generally informed by the owner or attendant that most all the horses in the stable suffered from pink-eye; but the one now lame escaped, or had a very mild attack.

Examination of such patients reveals the following:

The horse is lame to a greater or less extent in one limb. There is tenderness on pressure, increased heat, and redness of the skin (if the leg be white in color) in the region of the pastern and fetlock. The lameness increases in severity, and, at the expiration of two or three days, a serous discharge, somewhat yellowish in color, exudes through the skin and coagulates upon the hair which covers the parts.

About the fourth or fifth day, manipulation of the tender and swollen parts reveals fluctuations and a collection of fluid under the skin, resembling a large abscess. If cut into, or allowed to rupture without any surgical interference, a quantity of a redish-colored fluid escapes, mixed with shreds of devitalized areolar tissue.

If the wound is then explored, a large cavity will be found; the skin separated from the structures underneath to the extent of several inches. The skin itself, immediately over the part, seems to be dead, sinks on pressure, and presents a doughy feeling. Two or three days later a line of demarcation makes its appearance, separating the gangrenous from the living structures. The devitalized portion of the skin will slough out, leaving a wound several inches in diameter, with an irregular, granulating surface, which requires considable time to heal. In more aggravated cases the swelling and tenderness extend all the way up the limb, collections form in the cellular connective tissues, burrowing in between the deep-seated bloodvessels, nerves, ligaments and tendons, even through synovial capsules, into the cavities of joints.

In mild cases there is but little systemic disturbance, but in severe cases the constitutional effects are so severe as to destroy the animal's life. I have one case under my care at present, in which collections formed from the coronæ all the way up to the thigh, and there are at present nine openings in the leg, most of which communicate with each other, and all discharge large quantities of thin brownish-colored fluid, mixed with pus and shreds of connective tissue. The skin around these openings, to the extent of several inches, presents a sodden, doughy sensation to the touch, and to judge from present indications, the skin will probably slough from the larger portion of the leg.

The subject is a young horse, previous to present attack in good health, and escaped when most all the horses in the same stables suffered from pink-eye some three or four weeks ago. This horse is the property of Mr. Nugent, of Congress St., Brooklyn.

I saw him a few days after he showed the first signs of lameness, and an examination revealed considerable constitutional disturbance. Pulse increased in frequency, temperature elevated, loss of appetite, with redness and tenderness on pressure in the region of the pastern.

A cathartic was administered and a flaxseed poultice ordered to the inflamed pastern.

*Second day.*—General condition of patient about the same; lameness worse.

*Third day.*—Bowels acting freely; patient refuses all food; general condition about the same; the skin of the pastern had ruptured, and a copious discharge issued from the opening; the wound was cleansed with an antiseptic solution, the ragged edges removed, and ground flaxseed mixed with a strong solution of carbolic acid applied to the part.

*Fourth Day.*—Horse shows signs of extreme agony, held the affected limb up from the floor, sweat covered his body, pulse soft and very much increased in frequency—temperature 106. The leg is considerably swollen all the way up to the hock, and very sensitive to the touch. When poultice was removed from pastern, a large section of devitalized skin adhered to it, leaving an unhealthy granular surface beneath. Anodynes and sedatives were employed internally, the wounds dressed with antiseptics, and the upper part of the leg bathed with lead and opium wash.

*Fifth Day.*—Animal continued in great distress, lifting the affected limb constantly, breathing hurriedly, and the perspiration dropping off of him. Opium was given in full doses with aconite, wounds dressed with antiseptics, lead wash continued.

*Sixth Day.*—Distinct fluctuations could be detected in front of the fetlock; an opening was made which discharged freely; a cloth, saturated in a lead and opium solution, was applied to the metatarsal region, and kept wet constantly with the solution. The tincture of the chloride of iron was given in liberal doses.

*This morning*, on removing the cloth, seven or eight openings around the metatarsal bone, and one on the inner surface of the thigh, were exposed to view. All of them had ragged, dark-colored edges, and all discharged profusely. The animal was very much easier, but the general symptoms remain about the same. Temperature, 105; respiration, 80; pulse, 110.

At our next lecture, we will continue this subject; and I hope to be able to make a more favorable report upon this particular case, although his condition is at present extremely critical, I consider that there is still a chance for recovery.

